# The effect of retrograde material type and surgical techniques on the success rate of surgical endodontic retreatment: systematic review of prospective randomized clinical trials

**DOI:** 10.1186/s12903-021-01731-9

**Published:** 2021-07-24

**Authors:** Ashraf Abou ElReash, Hamdi Hamama, John C. Comisi, Ahmed Zaeneldin, Xie Xiaoli

**Affiliations:** 1 Department of Endodontic, Xiangya School of Stomatology, Central South University, Xiangya Road No 72. Kaifu, Changsha, 410078 Hunan Province China; 2 Department of Operative Dentistry, Faculty of Dentistry, Mansoura University, Mansoura, Egypt; 3 Department of Oral Rehabilitation, Medical University of South Carolina, James B. Edwards College of Dental Medicine, Charleston, SC USA; 4 Restorative Dental Sciences Department, Prince Philip Dental Hospital, The University of Hong Kong, Hong Kong, Hong Kong

**Keywords:** Apicectomy, Endodontic surgery, RCT, Retrograde filling

## Abstract

**Background:**

Endodontic surgical procedures, when performed, require retrograde filling materials that are biocompatible, non-toxic, non-irritant, dimensionally stable, and ideally promote bone formation. Precise evaluation of retrograde filling materials in clinical trials is necessary to give holistic view for properties of material and potential outcome from its use. The purpose of this review is to evaluate the effect of retrograde material type and surgical techniques on the success rate of surgical endodontic retreatment.

**Methods:**

An electronic search was performed in the time frame between 1st of January 2000 to 1st of September 2020 using database.

Sources

Web of Science, PubMed and redundant hand searches through their references. Seven inclusion–exclusion criteria were set for the selection and identification of relevant articles. Risk of bias was conducted for the included studies.

**Results:**

Nine randomized clinical trials (RCTs) fulfilled the inclusion criteria for this systematic review. The outcome of this review revealed that none of the reviewed trials totally-fulfilled CONSORT 2010 criteria.

**Conclusions:**

In light of the outcome of this review, there is no enough evidence to support the superiority of certain retrograde filling material or surgical technique over another in the success rate of surgical endodontics retreatment. The variety of methodologies and strategies, such as patient selection, the method of treatment and study analysis, led to doubtful credibility of the obtained clinical evidence. Further prospective randomized controlled clinical trials evaluating the specific effect of the various used materials are needed.

## Background

The ultimate goal of endodontic treatment is to achieve complete elimination of bacterial components and toxins during the mechanical shaping of the root canal system with a subsequent filling of that system with materials to provide a three-dimensional seal of that root canal system from coronal to apical aspects of that system [1, 2].

Despite the high success rates achieved with initial root canal treatment, endodontic failures can still occur [3]. The causality of failure can largely be attributed to bacterial invasion from inadequate cleansing of the root canal system, inaccessible areas when encountering complexities and irregularities of the root canal system, and from foreign body reactions such as extrusion of filling material and broken files or extra radicular biofilm [4, 5]. When there is non-surgical endodontic treatment failure, retreatment is typically considered, provided the tooth is restorable, and the canals are accessible. However, when non-surgical treatment cannot be achieved, surgical retreatment becomes a viable treatment modality [6].

Endodontic surgical procedures, when performed, require retrograde filling materials that are biocompatible, non-toxic, non-irritant, dimensionally stable, and ideally promote bone formation [7, 8]. Many different materials that have been suggested for use as a retrograde filling material, such as amalgam, composite resin, reinforced zinc oxide–eugenol cement (IRM; Dentsply), super ethoxybenzoic acid (Super-EBA; Bosworth Co, Skokie, IL) cement, and glass ionomer cement [9]. With such an array of choices, the clinician can encounter be considerable confusion as to which material would work best in the various clinical situations that they face. Precise evaluation of retrograde filling materials in clinical trials is necessary to give an accurate picture of the properties of the material and the potential outcome from its use.

Systematic reviews and meta-analytical studies are considered the highest level of evidence that supports evidence-based decision making, which is described as the ‘‘formalized process of using a specific set of skills for identifying, searching for and interpreting clinical and scientific evidence so that it can be used at the point of care”[10].

There are several prospective randomized clinical trials that assess the effect of root-end filling materials [11–21], And there are narrative reviews focused on the effect of the retrograde filling material itself. Assessment of the differences in the materials used, adopted techniques, and heterogeneity in studies of previous articles are required for a better understanding of surgical endodontic treatment protocols and to assess which variables affect clinical outcomes [22–25].

The systematic review by Pinto et al. [26] focused only one on evaluating only endodontic microsurgeries when used with different retrograde filling materials. Another systematic review by Seltzer et al. [23] compared endodontic microsurgery and tradition root-end surgery and concluded that there is a significant better prognosis in case of microsurgery. To date, there are no systematic reviews that evaluate the methodologies of prospective randomized clinical trials with a focus on the effect of different root-end filling materials. Hence, the evidence supporting the use of specific root-end filling material with a particular technique as a useful and efficacious technique remains weak. Accordingly, the objectives of this systematic review are to (1) assess the clinical outcome of using different root-end filling materials in previously published prospective randomized controlled clinical trials and (2) evaluate quality and extent of compliance of these studies with the requirements of ideal randomized clinical trials.

## Methods

The protocol for this systematic review was adopted from the PRISMA checklist for reporting systematic reviews [27] with registration number (20180029).

### Formulating review questions

A well-defined review question was developed by using the Patient Population, Intervention, Comparison, and Outcome (PICO) frame work to establish a systematic review of the current literature regarding radiographic outcomes of endodontic surgical retreatment in patients who have had prior endodontic treatment but have recurrent periapical pathosis and/or clinical symptoms.

- “P”—population—is the patients with teeth that were previously Endodontically treated and had periapical pathosis.
- “I”—the type of surgery (e.g., modern endodontic surgery).
- “C”—the comparison group: is the type of retrograde filling material used.
- “O”—the definition of outcome: according to the healing criteria of Rud et al./or Molven et al. [28, 29].

The critical questions of this systematic review were these:

1. What is the effect of using retrograde filling?
2. Does the use of specific material, device, or technique in surgery improve healing of the lesion or reduce patient post-operative discomfort?
3. Did the adopted methodologies in previously published studies fulfil the ideal requirements of a randomized clinical trial?

### Search methodology

An exhaustive broad literature search was done through three electronic databases, WEB OF SCIENCE, EL SEVIER, and PubMed searching for topic-related studies, regardless of the publication type using four keywords:

‘Endodontic surgery,’ ‘Retrograde filling,’ ‘Root end filling,’ ‘Apicectomy.’ The following combinations were used while searching the previously published literature; ‘Endodontic surgery AND retrograde filling,’ ‘Endodontic surgery AND root-end filling,’ ‘Apicectomy AND Retrograde filling,’ ‘Apicectomy AND Root end filling.’

Exploration of the literature was performed between the years 2000 and 2020 with an electronic database search and flow for article identification, screening process, and submission of the eligibility criteria with these articles. To identify ongoing or unpublished studies, personal contacts have also been used. Full articles were obtained for all the titles and abstracts (when available).

### Screening and data extraction

**Fig. 1:**
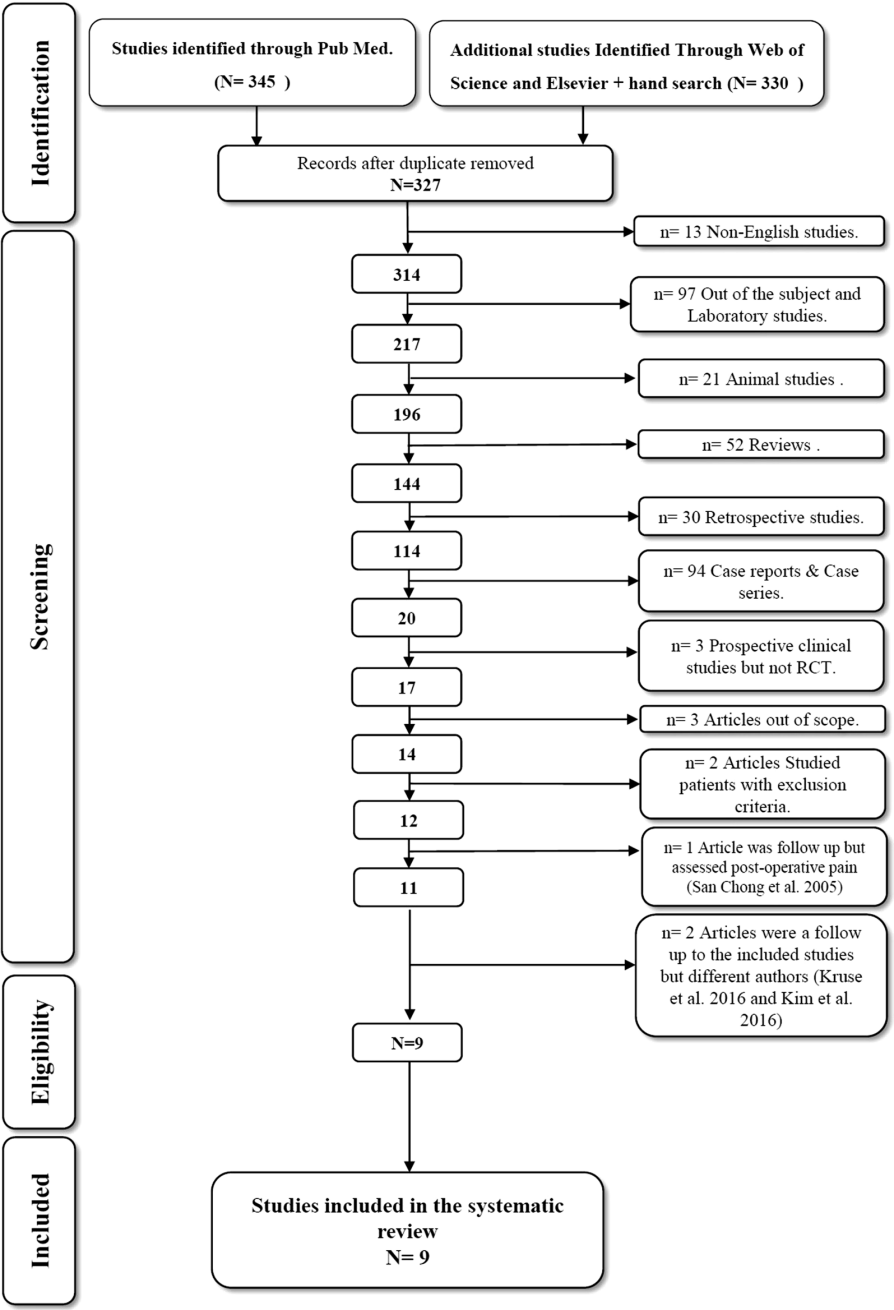
Prisma flow diagram

Initially, three reviewers analyzed the titles and abstracts of the resultant articles. The reviewers removed duplicate articles and completed an extensive hand search through the articles and references, searching for articles that hadn’t been found in the electronic search. The following categories were excluded during the assessment process: (1) non-English studies, (2) animal studies, (3) review articles, (4) laboratory studies, (5) case reports, and (6) non-randomized or retrospective clinical trials as illustrated in (Fig. 1).

All resulting titles and abstracts were screened independently in an unblinded standardized manner by the five reviewers for the relevance of the topic. Those abstracts meeting the inclusion criteria were selected, and the articles read in full. The approval of at least three reviewers was enough to include it in this systematic review. In case of no agreement, the final agreement was reached by discussion until a final decision was determined.

### Inclusion criteria

Inclusion criteria for this review included (1) articles in English, (2) prospective clinical trials with at least two arms, (3) studies with the follow-up period for a minimum of 12 months, (4) studies limited to humans, (5) informed consent must have been given to participants, (6) the sample size must be mentioned and (7) the outcomes (success and failure) evaluation must have been complying with the criteria of Rud et al./or Molven et al. [28, 29].

### Exclusion criteria

Exclusion criteria consisted of studies that did not meet the above inclusion criteria, which are: (1) non-randomized studies, non-English studies, (2) one arm studies, (3) lab or animal studies, (4) case reports, (5) reviews or opinion papers, (6) retrospective studies, (7) studies less than 12 months follow up, re-surgery studies, (8) detailed success rate not given or success rate calculation from raw data not possible, (9) use of guided tissue regeneration, (10) outcomes not evaluated according to the criteria above, (11) no sample design provided and (12) presence of a through and through lesion or a lesion of combined periodontal endodontic origin.

### Data extraction and quality assessment

Study quality and internal validity were assessed for each included trial according to the CONSORT 2010 checklist [30] by examining information such as ethical approval, study settings, sample size calculation, number of patients, type of teeth treated, number of surgeons, use of magnification (none, loupes, microscope), materials used, the age range of patients, follow-up period/intervals, and specific outcomes. These data were put into data extraction sheets, which were also used to evaluate information about elements of randomization, concealment of treatment allocation, blinding, and the handling of patient attrition.

### Risk assessment of selected studies

The same five reviewers independently assessed the risk of bias of included studies. Also, the final decision is reached by the approval of at least three reviewers. Disagreements were resolved after substantive discussion. The risk of bias assessment is built according to instructions provided by Higgins AND GREEN 2011 [31].

Five points were considered for each study: selection bias, performance bias, detection bias, attrition bias, and reporting bias. For every article, the risk was judged as low, unclear, or high. If a study had a low risk for each item, then it was judged to have a low risk of bias and a high level of evidence. If a study had an unclear risk for at least one item, but no item had a score at high risk, then the study is judged to have an unclear risk of bias and a moderate level of evidence. If a study had a high risk of bias at any of the previously mentioned items, the study is judged to be at high risk of bias and low level of evidence.

## Results and data analysis

### Excluded studies

The flow chart of the article screening process is presented in (Fig. 1). The electronic search and hand search gave after removal of duplicates, 327 records. After the screening of titles and abstracts, the following categories were excluded: 13 non-English studies, 97 out of subject studies and laboratory studies, 21 animal studies, 52 review articles, 30 retrospective studies, 94 case reports and case series, 3 prospective clinical studies but non-randomized and finally 17 full-text articles assessed for eligibility but 8 articles were excluded because 2 articles studied patient with exclusion criteria, 3 articles were out of our scope of research with different objectives, and 3 articles were a follow-up. We considered nine articles potentially eligible for inclusion.

In our analysis, eight articles didn’t meet our criteria for eligibility. The study of Pecora et al. [32] was excluded because the studied patients had exclusion criteria (through and through periradicular lesions) which will require bone grafting, while the study conducted by Da Silva et al. [33] had evaluation criteria of success and failure other than our inclusion criteria. The scope of the studies conducted by Taschieri et al., de Lange et al., Shearer et al. [34–36] aimed to test the effect of ultrasonic preparation and effect of magnification on periradicular surgery thus they were excluded. Meanwhile, Chong et al. [37] assessed mainly the post-operative pain after two years, in addition; the studies of Kim et al. and Kruse et al. [19, 20] were a follow up to previously included studies but were included in this systematic review.

### Characteristics of included studies

Nine studies met the inclusion criteria. The chosen randomized clinical trials had the following geographic distribution 3 trials in Sweden 33% Platt and Wannfors [13], Walivaara et al. [16], Walivaara et al. [17], 2 trials in Denmark 22% Jensen et al. [11]; Christiansen et al. [15], 1 trial in Netherlands 11% Lindeboom et al. [14], 1 trial in London 11% Chong et al. [12], 1 trial in china 11% Zhou et al. [21]and finally 1 trial was conducted in South Korea 11% Song and Kim–Kim et al. [18, 19] with total 77% of the trials in Europe and 22% in Asia.

Four studies (44%) clearly stated the funding sources, and 77% of the trials received ethical approval from relevant Institutional Review Boards (IRB). From the assessment of the methodologies, participant eligibility criteria were clearly stated in four studies, 40%, while the others were incompletely illustrated. The number of operators was as follows: (one) in four studies 44%, (two) in three studies 33%, (three) in one study 11%, and (four) in one study 11%. Concerning the blinding of evaluators, only five studies (55%) succeeded. Conversely, for blindness to treatment, no study managed to achieve it, neither the patients nor the operators.

Regarding sample size calculation, only 5 studies performed the analysis while the remaining studies did not clearly state this issue. In comparing long-term follow-up, 3 trials (33%) showed a follow-up period of more than one year. Variables such as type of the tooth, age, sex, and smoking, which can affect the results, were fully documented and included in only two studies (22%), while in 6 trials, it was not wholly illustrated. One study didn’t even discuss the topic. The size of the treated lesion was mentioned in four studies (44%), while other studies provided no information.

Four studies (45%) used no bevel or slight bevelling technique in root-end resection, while the other five studies (55%) indicated that the root apex was cut using traditional bevelling techniques. Magnification was used in 77% of the studies during surgical procedures ranging from loupes to operating microscope. The same studies also used ultrasonic for root-end cavity preparation. Furthermore, 44% of the included studies indicated the provided antibiotic prescriptions after surgery. At the same time, there is no mention of such, in the other studies, which may affect the results, as shown in Table 1.

**Table 1:** Illustration of information of included studies

	Jensen et al. [11]	Chong et al. [12]	Platt and Wannfors [13]	Lindeboom et al. [14]	Christiansen et al. [15] to Kruse et al. [20]	Walivaara etal. [16]	Walivaara et al. [17]	Song and Kim [18] and Kim et al. [19]	Zhou et al. [21]
Trial design:	RCT	RCT	RCT	RCT	RCT	RCT	RCT	RCT	RCT
Location:	Denmark	London	Sweden	Netherland	Denmark	Sweden	Sweden	Korea	China
Recruitment period:	?	?	?	1/2000 –12/2002	?	?	9/2006 –12/208	2/2003–10/2010	12/2012–2/2015
Source of funding:	?	√	?	√	√	?	?	√	?
Ethical approval	√	**√**	√	√	√	?	?	√	√
Informed consent	?	?	?	?	√	?	?	√	√
Eligibility criteria	IC	√	IC	√	IC	IC	IC	√	√
Number of surgeons	4	2	1	3	1	2	2	1	1
Blindness of the patients/operator/ evaluator	?/ × /√	?/?/√	× / × /?	√/?/√	?/ × /?	?/ × /?	?/ × /?	?/ × /√	?/?/√
Sample size	134Pt/134T	183 Pt/183T	28 Pt/34T	90Pt/100T	44Pt/52T	139Pt/160T	164 Pt/206T	260Pt/260T	240Pt/240T
S.S cal	√	√	×	**√**	×	×	** × **	√	√
After 1 year F. up	122Pt/122T	122 Pt/122 T	28 Pt/34 T	90 Pt/100 T	39 Pt/46 T	131pt/147 T	153pt/194 T	192Pt/192 T	120Pt/120 T
More than 1 years F. up	×	×	×	×	39 teeth 6 years	×	12–21 Month	182 4 years	×
Drop out	12Pt/12 T	61 Pt/61 T	×	×	5 Pt/6 T(1Y) 13 teeth (6Y)	8 Pt/13 T	9 Pt/12 T	68Pt (1Y) 10Pt (4Y)	82 Pt/82 T
Age at baseline	Age range of 49	?	33–83	17–64 Average 43.4	Range 30–77 years mean 54.6	Average 58.5	–	≤ 20 and ≥ 60 Frequency per age range	≤ 45 = 136, > 45 = 22
Gender	F/M ratio = 1.8:1	?	45% Female	57 F/33 M	24F/20 M	81 F/58 M	99 F/65 M	69 F/123 M	94 F/64 M
Smokers	48%	?	?	?	16/44	?	?	?	?
Clinical variables analysis	√	×	IC	×	√	×	IC	IC	√
Teeth treated	Max 27Ant 39Pm16 M Mand 6 Ant, 16Pm, 30 M	Ant teeth 1st PM, MB root of molar	Ant teeth	Single rooted ant teeth and PM	Max 17 incisors/24 canines and PM Mand 11 canines and PM	46 Incisor 10 Canine 42 PM 49 M	40 Incisor 16 Canine 57 PM 81 M	Max 73 Ant 31 PM 28 M Mand 21Ant, 11 PM, 28 M	Ant 113 PM 19 M 26
Size of lesion	< 5 mm = 52, 5–10 mm = 35, > 10 mm = 6	?	?	not > 10 mm	?	?	< 5 mm = 56, 5–9 mm = 102, > 9 mm = 36	?	Not > 10 mm
Use of magnification	×	√	×	√	√	√	√	√	**√**
Use of ultrasonic preparation	×	√	×	√	√	√	√	√	√
Angel of resection (bevelling)	√	×	√	√	×	√	√	×	×
Antibiotics prophylaxis	√	√	×	×	√	×	×	√	×
Comparison	Retro Plast CS GI	MTA versus IRM	Compomer Dyract versus Ketac Silver GI	MTA versus IRM	(MTA) versus smoothening of orthograde gutta-percha root filling	Ultrafill Gutta Percha versus IRM	IRM versus super EBA	MTA versus super EBA	MTA versus I root Bp plus

√ = yes, ? = not clear / not available, ×  = no, RCT = randomized clinical trial, IC = incomplete, S. Scal = sample size calculation, F. UP = follow up, MTA = mineral trioxide aggregate, IRM = intermediate restorative material, EBA = ethoxy benzoic acid CS = chelon silver GI = glass ionomer, Pt = patient, T = tooth, Max = maxillary, Mand = mandibular, ANT = anterior, PM = premolar, M = molar

The analysis of investigated materials mentioned in these studies illustrated: MTA (mineral trioxide aggregate) was tested in 55% of the trials, IRM (Intermediate restorative material) in 44%, Super EBA 22%, Ultrafill gutta-percha 11%, just smoothening of gutta-percha 11%, R.P. composite (Retroplast) 11%, C.S. Glass Ionomer (Chelon silver) 11%, Ketac silver Glass ionomer 11%, iRoot BP plus 11% and Compomer Dyract 11%. Assessment of the frequency of testing root-end filling materials in the studies was illustrated in Table 2.

**Table 2:** Frequency of test materials in included studies

	MTA	EBA	iRoot BP Plus	IRM	Ketac Silver	Compomer Dyract	Chelon-Silver	Retroplast Silver	GP
Number of studies tested	5	2	1	4	1	1	1	1	2
Percentage (%)	55	22	11	44	11	11	11	11	22

S, success; F, failure; Sig of Dif, significance of difference

In terms of success and failure, the outcome of each study for each material is illustrated in Table 3. Each one of the nine included studies compared two types of retrograde filling materials. MTA was evaluated against IRM in two studies Chong et al. and Lindeboom et al. [12, 14]. Both studies involved 222 teeth. Results after one year follow up showed no significant of difference between the two materials with no superiority of one material over the other. The same clinical results were noted when MTA was compared to Super EBA in the studies of Song and Kim, Kim et al. [18, 19] on 192 patients with no significant of difference between the two materials even after four years follow up. Also, the study of Zhou et al. [21] compared MTA with I Root BP Plus and conducted on 120 patients reported no significant of difference between the materials.

**Table 3:** Assessment of the outcomes of tested materials

Study	Materials																		Significance of difference
	MTA		Super EBA		iRoot BP Plus		IRM		Ketac silver		Compomer Dyract		Chelon silver		Retroplast		GP		
	S%	F%	S%	F%	S%	F%	S%	F%	S%	F%	S%	F%	S%	F%	S%	F%	S%	F%	
Jensen et al. [11]	–	–	–	–	–	–	–	–	–	–	–	–	52	48	82	18	–	–	Yes
Chong et al. [12]	84	16	–	–	–	–	76	24	–	–	–	–	–	–	–	–	–	–	No
Platt and Wannfors [13]	–	–	–	–	–	–	––	–	44	56	89	11	–	–	–	–	–	–	Yes
Lindeboom et al. [14]	92	8	–	–	–	–	86	14	–	–	–	–	–	–	–	–	–	–	No
Christiansen et al. [15]	96	4	–	–	–	–	–	–	–	–	–	–	–	–	–	–	52	48	Yes
Wälivaara et al. [16]	–	–	–	–	–	–	84.8	15.2	–	–	–	–	–	–	–	–	89.6	10.4	No
Wälivaara et al. [17]	–	–	81.6	18.4	–	–	90.6	9.4	–	–	–	–	–	–	–	–	–	–	No
Song et al. [43]	95.6	4.4	93.1	6.9	–	–	–	–	–	–	–	–	–	–	–	–	–	–	No
Kim et al. [19]-4 years (follow up)	91.6	8.4	89.9	10.1	–	–	–	–	–	–	–	–	–	–	–	–	–	–	No
Kurse et al. [20]–6 years (follow up)	84	16	–	–	–	–	–	–	–	–	–	–	–	–	–	–	55	45	Yes
Zhou et al. [21]	93.1	6.9	–	–	94.4	5.6	–	–	–	–	–	–	–	–	–	–	–	–	No

When MTA was compared against gutta-percha (GP) in the study by Christiansen et al. [15] on 46 teeth, followed up six years later by Kurse et al. [20], MTA showed superiority and significant of difference over gutta-percha. Nevertheless, IRM showed no superiority when compared to either GP or Super-EBA in two studies, Walivaara et al. [16, 17], respectively, with no significant of difference. Retroplast composite resin showed superior results when compared to Chelon silver in the study of Jensen et al. [11] on 134 participants with higher significance; also, Dyract compomer had superior results against Ketac Silver in the study of Platt and Wannfors [13].

### Risk of bias analysis

After analyzing the data, only one study, Lindeboom et al. [14] was judged to be at low risk of bias while two studies Christiansen et al. [15] and Zhou et al. [21] were judged to be at unclear risk. The remaining six studies Jensen et al., Chong et al., Platt and Wannfors, Wälivaara et al., Wälivaara et al. and Song and Kim, Kim et al. [11–13, 16–18] are considered to be at high risk of bias. So, we have, according to the level of evidence, 66% of the studies had a high risk of bias, 22% moderate risk, and only 11% had a low risk of bias.

**Fig. 2:**
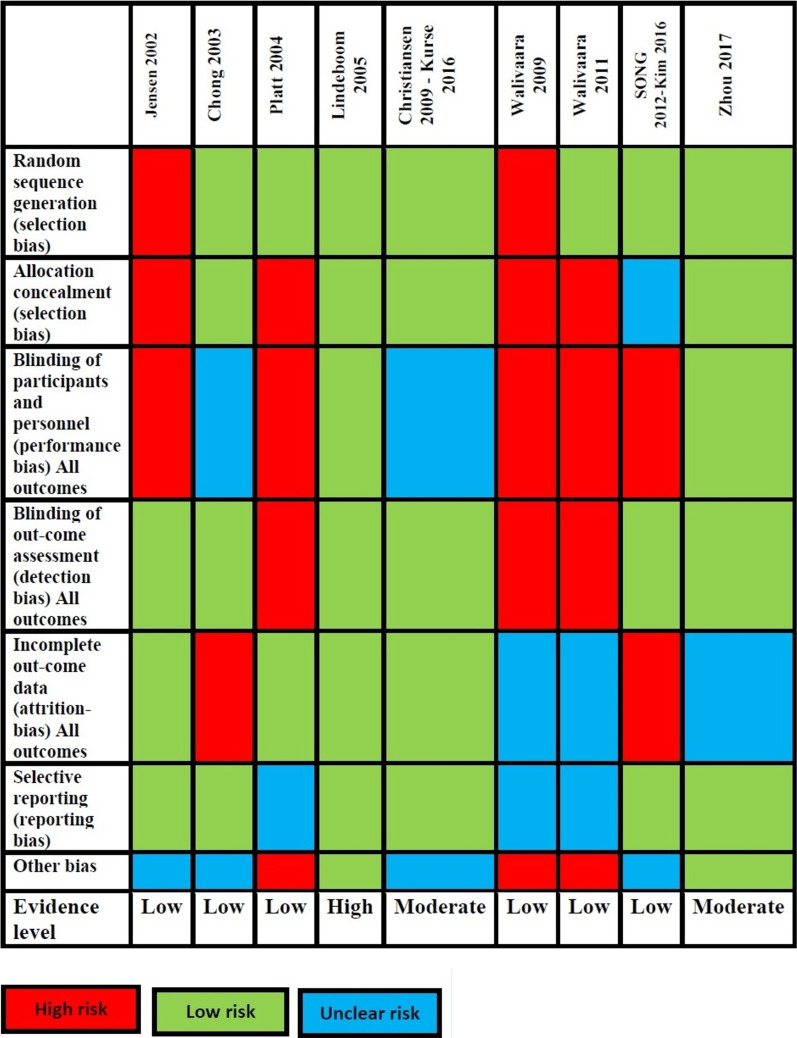
Risk of bias of included studies

Through all studies, it seems that random sequence bias had a 22% high risk of bias and 77% low risk of bias. Meanwhile, allocation concealment took 44% high risk of bias and 11% unclear risk of bias and 44% low risk of bias. Regarding blinding of participants was the highest risk of bias with 55% between studies and 22% unclear risk and lowest percent 22% for low risk of bias. Conversely, in the blinding of outcome assessment, high risk took 33%, and low risk took 66%. For attrition bias 44% low risk, 33% for unclear risk, and 22%for high risk of bias. Likely in reporting bias, we found 66% for low risk and 33% for unclear risk. Lastly, for other types of bias, the highest percentage was for unclear bias 44% and 33% for high risk and 22% for low risk of bias, as shown in (Fig. 2).

## Discussion

After reviewing all the previous randomized clinical trials, no clinical trial has completely fulfilled the requirements of the CONSORT 2010 checklist for quality assessment of randomized clinical trials [30]. The clinical trials performed by Lindeboom et al. and Zhou et al. are the only clinical trials that achieved accepted percent from the required items of the checklist including: demonstration of the ethical approval, eligibility criteria, sample size calculation, number of surgeons and evaluators, the blinding of evaluators to treatment, number and reasons of drop out of patients and use of modern tools in performing the operations [14, 21].

Throughout the studies analyzed, MTA was the most frequently compared material with other filling materials. Four studies showed no significant difference in success rates between the use of MTA and IRM as a retrograde filling material for the first two articles and super EBA in the third and iRoot BP plus in the fourth respectively [12, 14, 18, 21]. Only one study showed significant difference when using MTA in comparison with Gutta Percha. [15, 20] The study of Jensen et al. revealed a higher success rate with significant difference of Retroplast over Chelon silver [11]. Platt and Wannfors study also reported superiority of Compomer over Ketac Silver [13]. Meanwhile, IRM showed no significant difference when compared to Gutta Percha [16]. It was surprising that the same results obtained by Walivaara et al. [17] when compared IRM to Super EBA, which lead to doubtful clinical evidence.

It should be noted that missing information can influence the success rate analysis of clinical variables, including the type and status of the teeth being treated, quality of previous root filling, patient status such; as age, sex, smoking, and alcohol consumption. The techniques and parameters followed can affect the treatment outcome, and it is important to be equally distributed among the participants [22].

Regarding the lesion size Kim [38] stated that the size of the apical lesion is a significant determinant of treatment outcome, Jansson et al. [39] also found poor prognosis with larger periapical lesions—however, Grung et al. [40] found no relationship between lesion size and prognosis. Only four of the viewed trials mentioned the size of the lesion, while six did not. Lindeboom et al. [14] and Zhou et al. [21] included patients with lesion size not exceeding 10 mm. This inclusion can affect the results because, according to Christiansen et al. rate of successful healing was attributed to the size of the lesion; the smaller the lesion, the higher the rate of success [15].

The correlation between the type of the tooth being treated and the success and failure rates is not clearly defined yet. Many articles reported higher success rates associated with anterior teeth after endodontic surgery, which may be attributed to more accessibility and more precise visualization of the operating field, all these factors lead to better handling and improved apical seal attainment [41–43]. These factors may explain the success rate results of Platt and Wannfors and Christiansen et al. [13, 15].

Most of the investigators adopted modern techniques, including magnification devices, like loups and microscope to improve the visualization during the treatment. Also, they used microsurgical ultrasonic tips for preparation which have been proven to be faster compared to rotary burs and require less bone removal [44]. Jensen et al. [11] and Platt and Wannfors [13] did not adopt modern techniques but used the traditional root-end resection with a bur. It was not clinically proved yet the difference in the rate of lesions healing of treated cases using either loupes or surgical microscope except in improvement in the visualization and illumination of the surgical field, details of the apical part of the root, and more conservative bone removal [38, 45, 46].

The combination of magnification with modern techniques of root-end resection and ultrasonic preparation allows for more conservative preparations, less exposure of dentinal tubules, more efficient disinfection, and more effective adaptation and sealing of the retrograde filling to the root canal walls and consequently increased chances for higher success rates [23].

It would not be appropriate to conclude that healing/success would be based solely on clinical and radiographic outcomes after a follow-up period of only one year, ignoring that success versus failure might be influenced by an extended follow-up period. Moreover, judging lesions that failed to decrease in size during this period might show improvement with longer follow up periods and thereby become successful [47].

By assessing the risk of bias, via random sequence generation, we considered the randomization methods followed are accepted in all trials and considered to be at low risk except in Jensen et al. [11] and Wälivaara et al. [16] which were considered to be at high risk of bias. The methods used were not reported in Jensen et al. [11], while in Walivaara et al. [16], patients were assigned according to their date of birth. We found that allocation concealment was not stated or attempted in Jensen et al. [11] and Platt and Wannfors [13]. Therefore; the studies of Wälivaara et al. [16, 17] were considered to be at high risk of bias, while Song and Kim [18] and Song et al. [43] was unclear at this point. We considered the studies of Chong et al. [12]; Lindeboom et al. [14]; Christiansen et al. [15] and Zhou et al. [21] have adequate allocation concealment methodology. Furthermore, the presence of different follow-up investigators as in the studies of Christiansen et al. [15]; Kruse et al. [20] and Song and Kim [18]; Kim et al. [19] decreased the risk of bias.

Our study has several limitations, starting with the exclusion of non-English studies, prospective studies, but lack of randomization and short follow up period studies. The quality of the selected RCTs varied. Randomization was adequate in most trials; however, analyses did not identify a correlation between retrograde filling type and surgical technique, and favorable healing outcomes.

Publication bias might appear in some trials regarding the allocation concealment, participant, and outcome assessment. Larger trials, generally, analyzed with more methodological rigor than smaller ones, and through methodological assessment of all trials suggest that shortage of reporting in some items may have led to an overestimation of the effect of some retrograde fillings. In addition, the applicability of this review might be affected because there are no data for other populations in the world where the intervention might perform differently.

## Conclusions

The current scientific evidence shows that none of the used materials was significantly better than the other in clinical application except when comparing glass ionomer cement to composite materials or MTA to gutta-percha. The variety of methodologies and strategies, starting from patient selection to the method of treatment and analysis, led to doubtful credibility of the obtained clinical evidence. Further prospective randomized controlled clinical trials evaluating the specific effect of the various used materials and following the ideal requirements of clinical trials are needed.

## Data Availability

All data generated or analyzed during this study are included in this published article.

## Abbreviations

ANT: Anterior
CS: Chelon silver
F. UP: Follow up
GI: Glass ionomer
GP: Gutta percha
IC: Incomplete
IRM: Intermediate restorative material
M: Molar
Max: Maxillary
Mand: Mandibular
MTA: Mineral trioxide aggregate
Pt: Patient
PICO: Patient population, intervention, comparison, and outcome
PM: Premolar
RCT: Randomized clinical trials
S. S Cal: Sample size calculation
Super EBA: Super ethoxybenzoic acid
T: Tooth

## References

[1] Spångberg LS, Haapasalo M (2002). Rationale and efficacy of root canal medicaments and root filling materials with emphasis on treatment outcome. Endod Topics.

[2] Torabinejad M, Chivian N (1999). Clinical applications of mineral trioxide aggregate. J Endod.

[3] Friedman S (2002). Considerations and concepts of case selection in the management of post-treatment endodontic disease (treatment failure). Endod Topics.

[4] Torabinejad M, Watson T, Ford TP (1993). Sealing ability of a mineral trioxide aggregate when used as a root end filling material. J Endod.

[5] Foster KH, Harrison E (2008). Effect of presentation bias on selection of treatment option for failed endodontic therapy. Oral Surg Oral Med Oral Pathol Oral Radiol Endod.

[6] Gutmann JL, Harrison JW (1991). Surgical endodontics.

[7] Bodrumlu E (2008). Biocompatibility of retrograde root filling materials: a review. Aust Endod J.

[8] Costa A, Post L, Xavier C, Weber J, Gerhardt-Oliveira M (2008). Marginal adaptation and microleakage of five root-end filling materials: an in vitro study. Minerva Stomatol.

[9] Keiser K, Johnson CC, Tipton DA (2000). Cytotoxicity of mineral trioxide aggregate using human periodontal ligament fibroblasts. J Endod.

[10] Forrest JL (2009). Evidence-based decision making: a translational guide for dental professionals.

[11] Jensen S, Nattestad A, Egdø P, Sewerin I, Munksgaard E, Schou S (2002). A prospective, randomized, comparative clinical study of resin composite and glass ionomer cement for retrograde root filling. Clin Oral Investig.

[12] Chong B, Pitt Ford T, Hudson M (2003). A prospective clinical study of Mineral Trioxide Aggregate and IRM when used as root-end filling materials in endodontic surgery. Int Endod J.

[13] Platt AS, Wannfors K (2004). The effectiveness of compomer as a root-end filling: a clinical investigation. Oral Surg Oral Med Oral Pathol Oral Radiol Endod.

[14] Lindeboom JA, Frenken JW, Kroon FH, van den Akker HP (2005). A comparative prospective randomized clinical study of MTA and IRM as root-end filling materials in single-rooted teeth in endodontic surgery. Oral Surg Oral Med Oral Pathol Oral Radiol Endod.

[15] Christiansen R, Kirkevang LL, Hørsted-Bindslev P, Wenzel A (2009). Randomized clinical trial of root-end resection followed by root-end filling with mineral trioxide aggregate or smoothing of the orthograde gutta-percha root filling–1-year follow-up. Int Endod J.

[16] Wälivaara D-Å, Abrahamsson P, Sämfors K-A, Isaksson S (2009). Periapical surgery using ultrasonic preparation and thermoplasticized gutta-percha with AH Plus sealer or IRM as retrograde root-end fillings in 160 consecutive teeth: a prospective randomized clinical study. Oral Surg Oral Med Oral Pathol Oral Radiol Endod.

[17] Wälivaara D-Å, Abrahamsson P, Fogelin M, Isaksson S (2011). Super-EBA and IRM as root-end fillings in periapical surgery with ultrasonic preparation: a prospective randomized clinical study of 206 consecutive teeth. Oral Surg Oral Med Oral Pathol Oral Radiol Endod.

[18] Song M, Kim E (2012). A prospective randomized controlled study of mineral trioxide aggregate and super ethoxy–benzoic acid as root-end filling materials in endodontic microsurgery. J Endod.

[19] Kim S, Song M, Shin S-J, Kim E (2016). A randomized controlled study of mineral trioxide aggregate and super ethoxybenzoic acid as root-end filling materials in endodontic microsurgery: long-term outcomes. J Endod.

[20] Kruse C, Spin-Neto R, Christiansen R, Wenzel A, Kirkevang L-L (2016). Periapical bone healing after apicectomy with and without retrograde root filling with mineral trioxide aggregate: a 6-year follow-up of a randomized controlled trial. J Endod.

[21] Zhou W, Zheng Q, Tan X, Song D, Zhang L, Huang D (2017). Comparison of mineral trioxide aggregate and iRoot BP plus root repair material as root-end filling materials in endodontic microsurgery: a prospective randomized controlled study. J Endod.

[22] Del Fabbro M, Corbella S, Sequeira‐Byron P, Tsesis I, Rosen E, Lolato A, Taschieri S. Endodontic procedures for retreatment of periapical lesions. Cochrane Database Syst Rev*.* 2016(10). 10.1002/14651858.CD005511.pub3 PMC6461161 27759881

[23] Setzer FC, Shah SB, Kohli MR, Karabucak B, Kim S (2010). Outcome of endodontic surgery: a meta-analysis of the literature—part 1: comparison of traditional root-end surgery and endodontic microsurgery. J Endod.

[24] Tsesis I, Rosen E, Taschieri S, Strauss YT, Ceresoli V, Del Fabbro M (2013). Outcomes of surgical endodontic treatment performed by a modern technique: an updated meta-analysis of the literature. J Endod.

[25] García-Guerrero C, Guauque SQ, Molano N, Pineda GA, Nino-Barrera JL, Marín-Zuluaga DJ (2017). Predictors of clinical outcomes in endodontic microsurgery: a systematic review and meta-analysis. G Ital Endod.

[26] Pinto D, Marques A, Pereira JF, Palma PJ, Santos JM (2020). Long-term prognosis of endodontic microsurgery—a systematic review and meta-analysis. Medicina (Kaunas).

[27] Liberati A, Altman DG, Tetzlaff J, Mulrow C, Gøtzsche PC, Ioannidis JP, Clarke M, Devereaux PJ, Kleijnen J, Moher D (2009). The PRISMA statement for reporting systematic reviews and meta-analyses of studies that evaluate health care interventions: explanation and elaboration. J Clin Epidemiol.

[28] Rud J, Andreasen J, Jensen JM (1972). A follow-up study of 1,000 cases treated by endodontic surgery. Int J Oral Surg.

[29] Molven O, Halse A, Grung B (1987). Observer strategy and the radiographic classification of healing after endodontic surgery. Int J Oral Maxillofac Surg.

[30] Moher D, Hopewell S, Schulz KF, Montori V, Gøtzsche PC, Devereaux P, Elbourne D, Egger M, Altman DG (2010). CONSORT 2010 explanation and elaboration: updated guidelines for reporting parallel group randomised trials. J Clin Epidemiol.

[31] Higgins JP, Green S (2011). Cochrane handbook for systematic reviews of interventions.

[32] Pecora G, De Leonardis D, Ibrahim N, Bovi M, Cornelini R (2001). The use of calcium sulphate in the surgical treatment of a ‘through and through’periradicular lesion. Int Endod J.

[33] Silva SRD, Neto S, Schnaider TB, Veiga DF, Novo NF, Mesquita Filho M, Ferreira LM (2016). The use of a biocompatible cement in endodontic surgery: a randomized clinical trial 1. Acta Cir Bras.

[34] de Lange J, Putters T, Baas EM, van Ingen JM (2007). Ultrasonic root-end preparation in apical surgery: a prospective randomized study. Oral Surg Oral Med Oral Pathol Oral Radiol Endod.

[35] Shearer J, McManners J (2009). Comparison between the use of an ultrasonic tip and a microhead handpiece in periradicular surgery: a prospective randomised trial. Br J Oral Maxillofac Surg.

[36] Taschieri S, Del Fabbro M, Testori T, Francetti L, Weinstein R (2006). Endodontic surgery using 2 different magnification devices: preliminary results of a randomized controlled study. J Oral Maxillofac Surg.

[37] San Chong B, Ford TRP (2005). Postoperative pain after root-end resection and filling. Oral Surg Oral Med Oral Pathol Oral Radiol Endod.

[38] Kim S, Kratchman S (2006). Modern endodontic surgery concepts and practice: a review. J Endod.

[39] Jansson L, Sandstedt P, Låftman A-C, Skoglund A (1997). Relationship between apical and marginal healing in periradicular surgery. Oral Surg Oral Med Oral Pathol Oral Radiol Endod.

[40] Grung B, Molven O, Halse A (1990). Periapical surgery in a Norwegian county hospital: follow-up findings of 477 teeth. J Endod.

[41] Peñarrocha M, Martí E, García B, Gay C (2007). Relationship of periapical lesion radiologic size, apical resection, and retrograde filling with the prognosis of periapical surgery. J Oral Maxillofac Surg.

[42] von Arx T, Penarrocha M, Jensen S (2010). Prognostic factors in apical surgery with root-end filling: a meta-analysis. J Endod.

[43] Song M, Jung I-Y, Lee S-J, Lee C-Y, Kim E (2011). Prognostic factors for clinical outcomes in endodontic microsurgery: a retrospective study. J Endod.

[44] Palma PJ, Marques JA, Casau M, Santos A, Caramelo F, Falacho RI, Santos JM (2020). Evaluation of root-end preparation with two different endodontic microsurgery ultrasonic tips. Biomedicines..

[45] Taschieri S, Del Fabbro M, Testori T, Weinstein R (2007). Efficacy of xenogeneic bone grafting with guided tissue regeneration in the management of bone defects after surgical endodontics. J Oral Maxillofac Surg.

[46] Setzer FC, Kohli MR, Shah SB, Karabucak B, Kim S (2012). Outcome of endodontic surgery: a meta-analysis of the literature—part 2: comparison of endodontic microsurgical techniques with and without the use of higher magnification. J Endod.

[47] Iqbal MK, Kim S (2008). A review of factors influencing treatment planning decisions of single-tooth implants versus preserving natural teeth with nonsurgical endodontic therapy. J Endod.

